# Mesenchymal Stem Cells: Characteristics, Function, and Application

**DOI:** 10.1155/2019/8106818

**Published:** 2019-03-06

**Authors:** Zhongjun Li, Xingbin Hu, Jiang F. Zhong

**Affiliations:** ^1^Department of Blood Transfusion, Lab of Radiation Biology, The Second Affiliated Hospital, Army Medical University, Chongqing, China; ^2^Department of Blood Transfusion, The First Affiliated Hospital, Fourth Military Medical University, Xi'an, China; ^3^Division of Biomedical Sciences, University of Southern California, Los Angeles, CA, USA; ^4^Division of Periodontology, Diagnostic Sciences & Dental Hygiene, School of Dentistry, University of Southern California, Los Angeles, CA, USA

Adult mesenchymal stem cells (MSCs) are widely regarded as a plastic-adherent cell population, which has self-renewal capacity and can be differentiated into osteogenic, chondrogenic, adipogenic, and other lineages [1, 2]. In addition, MSCs showed low immunogenicity and strong immunomodulation potential [3]. MSCs can be isolated and identified from many tissues, such as bone marrow, adipose tissue, dermal tissue, intervertebral disc, amniotic fluid, various dental tissues, human placenta, and cord blood [4]. MSCs have functions in the maintenance of tissue homeostasis and have potential applications in regenerative medicine. These cells have been successfully employed in clinical applications, such as cartilage and bone repair, skin wound healing, neuronal regeneration, heart regeneration, and immune disorder treatment including treatment of graft versus host disease (GvHD) due to their abilities of “homing,” multilineage differentiation, and immunomodulation [5]. However, the natural characteristics and function of MSCs from different tissues are heterogeneous [6]; therefore, deeper understanding of the differences of MSCs derived from various sources would help us to make better choices for disease treatment. Furthermore, the culture expansion *in vitro* has been reported to attenuate the homing ability of MSCs [7], which suggests that MSCs may lose some of their natural characteristics after expansion *in vitro*. Hence, novel strategies for maintaining, and even promoting, the biological activities and therapeutic capabilities of MSCs are really needed.

In this special issue, we have invited state-of-the-art research contributions on MSC characteristics, functions, and applications. After a rigorous review process, we selected seven papers to appear here. Y. Ma et al. isolated and identified MSC-like cells expressing alpha-smooth muscle actin (*α*-SMA) from adult human sweat glands (ahSGs) for the first time. These cells are located in the basal myoepithelial areas of the secretory portion of the solenoid bulb. This report demonstrated that there are tissue-specific MSCs in ahSGs that may contribute to wound repair and sweat gland regeneration. C.A.F. Mançanares et al. found that yolk sac-derived MSCs could form tubular structures *in vitro*, which suggested the possible formation of blood capillaries *in vitro* by MSCs. T. Khatlani et al. have assessed the biological activities of MSCs from the decidua basalis (DBMSCs) after exposing DBMSCs to hydrogen peroxide (H_2_O_2_). The results in their study indicated that DBMSCs could resist an oxidative stress environment and gain beneficial effects from this toxic environment on their ability to repair injured tissues. J. Zhan et al. reported that fasudil, a potent rho kinase inhibitor, significantly enhanced the migration ability of bone marrow MSCs via activation of the MAPK signaling pathway, promoting MSC homing to the spinal cord injury site. This study showed that combination of MSC transplantation and fasudil administration might have beneficial effects in therapy after central nervous system trauma. Y. Nakashima et al. performed a protein expression analysis by liquid chromatography with tandem mass spectrometry (LC-MS/MS) to investigate the influences of cell stress resulting from passage on protein expression in adipose-derived MSCs (AT-MSCs). They found that AT-MSCs retained their cell properties after three passages but showed a decreased protein expression. In addition, there are two reviews in this special issue. A. Tomokiyo et al. reviewed the current understanding of the features and functions of MSCs in periodontal ligament (PDL) tissue and discussed their potential applications for PDL regeneration. The review by X. Ambriz et al. focused on the mechanobiology of the actin cytoskeleton in stem cells, which is important in stem cell differentiation and interaction with biomaterials.

These studies in this special issue span a wide range, discussing the new organizational sources, biological activities, mechanobiology and proteomic profiling of MSCs. We believe that these studies have the potential to make great contributions to the research and clinical applications of MSCs.

Finally, we would like to express our deep gratitude to the reviewers for their valuable contributions to this special issue.
